# Lancing the Gums

**Published:** 1879-01

**Authors:** 


### ARTICLE VII.

### *Lancing the Gums.*

Prof. Gibbons, in an address before the Graduating Class of the Pacific Medical College, which is published in the *Pacific Medical & Surgical Journal*, says:

"Among the 'lost arts,' there is one the abandonment of which I condemn without qualification. I refer to the cutting of the gums in dentition. In discarding this simple expedient, our profession have thrown away a safe and valuable adjuvant in the management of infantile disorders. The only objections to it are that it gives pain, that it hardens the gums so as to retard the advance of the tooth, and that it endangers hemorrhage. So far from giving pain, it relieves pain, and more still the intolerable itching which children suffer while teething. If hardness result from the cicatrization, it will facilitate the escape of the tooth instead of retarding it; for every tyro knows that a cicatrix is absorbed under pressure more readily than normal tissue. And as to the danger from hemorrhage, fifty years of constant and abundant experience in my own practice, and observation of the practice of others around me when the operation was universal, have failed to bring within my knowledge a solitary instance of serious hemorrhage caused by lancing the gums. On the other hand, again and again have I seen the infant, when fretting and twitching and starting as if on the brink of a convulsion, fall into a tranquil sleep immediately after the process. More than once have I known the child close its jaw to press the lancet into the itching. One child I remember who would run to meet me as I entered the house and open its mouth to invite what experience had taught it, would relieve its suffering.

By lancing the gums I do not mean slicing off the prominence, nor yet making a crucial incision. These are superfluous, if not barbarous procedures. It is sufficient to scarify the swollen tissue in one direction, to relieve the tension and remove a few drops of blood from the engorged vessels."

